# Epitranscriptome-wide profiling identifies RNA editing events regulated by ADAR1 that are associated with DNA repair mechanisms in human TK6 cells

**DOI:** 10.3389/fgene.2025.1663827

**Published:** 2025-10-15

**Authors:** Akito Yoshida, Yuqian Song, Hotaru Takaine, Sujin Song, Nonoka Konishi, Yu-Hsien Hwang-Fu, Zachary Johnson, Kiyoe Ura, Akira Sassa

**Affiliations:** ^1^ Department of Biology, Graduate School of Science, Chiba University, Chiba, Japan; ^2^ Alida Biosciences Inc., San Diego, CA, United States

**Keywords:** A-to-I editing, ADAR1, DNA repair, epitranscriptome, TK6, DNA damage

## Abstract

**Introduction:**

Adenosine-to-Inosine (A-to-I) editing is an endogenous RNA modification in eukaryotes, catalyzed by adenosine deaminases acting on RNA (ADARs). This modification modulates the gene expression by influencing splicing, RNA stability, and coding potential, depending on the site of editing. Although recent studies suggest a crosstalk between A-to-I editing and transcripts involved in DNA repair, the extent and functional significance of this interaction remain unclear.

**Methods:**

To investigate this, we applied the EpiPlex RNA assay—a method enabling epitranscriptome-wide detection of RNA modifications—in human lymphoblastoid TK6 cells.

**Results:**

Across two biological replicates, we identified 870 transcripts bearing A-to-I–modifications. Gene Ontology analysis revealed significant enrichment in genome maintenance pathways, including “chromatin remodeling” and “DNA repair.” Notably, 27 transcripts encoding DNA repair proteins—such as *ATM, FANCA, BRCA1, POLH,* and *XPA*—contained A-to-I sites within introns or 3′ untranslated regions. To assess the isoform-specific contributions of ADAR enzymes—specifically ADAR1 p150 and p110—to RNA editing, we generated p150-deficient (p150 KO) and p150/p110-deficient (p150/p110 KO) TK6 cells. A-to-I editing peaks were reduced by ∼73.4% in p150 KO cells and nearly abolished (99.9%) in p150/p110 KO cells, indicating that most editing sites are p150-dependent, while a notable subset relies on p110. Importantly, a novel splice variant of *XPA* emerged in ADAR1-deficient cells, suggesting a role for RNA editing in alternative splicing regulation.

**Discussion:**

Our epitranscriptomic analysis of A-to-I RNA editing underscores a multifaceted role for ADAR1-dependent editing in preserving genome integrity through post-transcriptional regulation of DNA repair genes, laying the groundwork for future studies into RNA-based mechanisms of genome maintenance.

## 1 Introduction

RNA modifications are post-transcriptional chemical alterations of RNA nucleobases that play crucial roles in gene regulation in mammals. Major RNA modifications such as *N* ^6^-methyladenosine (m6A), 5-methylcytidine (5-mC), pseudouridine (Ψ), Adenosine-to-Inosine (A-to-I) editing, dynamically regulate RNA metabolism, including splicing, nuclear export, stability, translation, and degradation (Cui L. et al., 2022). Among these, A-to-I editing is a unidirectional RNA modification catalyzed by Adenosine Deaminase Acting on RNA (ADAR), which specifically convert adenosine-to-inosine within double-stranded RNA (dsRNA) regions (Wang et al., 2017). In humans, the ADAR family comprises three members: *ADAR1, ADAR2*, and the catalytically inactive *ADAR3*. While *ADAR2* is predominantly expressed in the central nervous system, *ADAR1* is broadly expressed across various tissues. ADAR1 exists in two major isoforms generated through alternative splicing: the constitutively expressed nuclear isoform *ADAR1* p110, and the interferon-inducible cytoplasmic isoform *ADAR1* p150, which contains a Z-DNA-binding domain (George and Samuel, 1999; Sun et al., 2021). Notably, ADAR1 p150 prevents inappropriate activation of innate immune signaling pathways by editing cytoplasmic dsRNAs (Liddicoat et al., 2015; Chung et al., 2018; Nakahama et al., 2021). In addition to its enzymatic activity, ADAR1 also functions as an RNA-binding scaffold protein independent of A-to-I-editing. For instance, it directly interacts with Dicer to facilitate pre-miRNA processing and enhance miRNA loading into the RNA-induced silencing complex, highlighting its role in RNA interference (Ota et al., 2013). Furthermore, ADAR1 p110 has been shown to stabilize antiapoptotic mRNAs following UV irradiation by inhibiting Staufen1-mediated mRNA decay, thereby supporting cell survival (Sakurai et al., 2017).

Advancements in next-generation sequencing technologies have enabled transcriptome-wide identification of A-to-I editing sites (Mansi et al., 2021; Picardi et al., 2017). The functional impact of A-to-I editing varies depending on the location of the modification within the mRNA (Yuan et al., 2023). A significant proportion of editing occurs in the 3′ untranslated regions (3′UTRs) and introns, where dsRNA structures formed by Alu repeat elements serve as editing substrates (Ouyang et al., 2018; Qiu et al., 2016; Athanasiadis et al., 2004). Intronic editing can influence alternative splicing by creating or abolishing splice sites, as observed in genes such as *NARF* and *CDDC15* (Lev-Maor et al., 2007; Tang et al., 2020). Editing within 3′UTRs can disrupt miRNA binding, thereby modulating mRNA stability and translational efficiency. For example, ADAR1-mediated editing of *DHFR* and *METTL3* mRNAs has been linked to increased expression of these genes and tumor progression in breast cancer cells (Nakano et al., 2017; Li et al., 2022). A-to-I editing can also occur within coding regions, leading to amino acid substitutions that may alter protein function. ADAR2-mediated editing of the AMPA receptor subunit GluA2 exemplifies this, as it controls calcium permeability and suppresses excitotoxicity in neurons (Sommer et al., 1991). Additionally, A-to-I editing of lncRNAs can affect RNA secondary structure and miRNA binding, thereby regulating gene expression. In the case of NEAT1, such modifications enhance RNA folding stability and prevent competition with miR-9-5p, ultimately modulating JAK-STAT signaling and interferon-responsive genes expression (Chattopadhyay et al., 2024).

Recent studies have proposed that A-to-I editing plays a regulatory role in the DNA damage response and DNA repair pathways. For example, ADAR1-mediated editing within the exon of *NEIL1* mRNA, which encodes a DNA glycosylase, results in a K242R amino acid substitution. The edited NEIL1 protein exhibits diminished capacity to recognize and excise damaged bases (Yeo et al., 2021; Lotsof et al., 2022). A-to-I editing has also been reported in the 3′UTRs of *BRCA2, ATM*, and *POLH* mRNAs, where increased editing correlates with transcript expression levels (Sagredo et al., 2018). In the case of *BRCA2,* editing prevents miRNA binding, thereby stabilizing the mRNA and enhancing protein levels, contributing to cisplatin resistance (Liu et al., 2024). These findings highlight the need for systematic investigation into the interplay between A-to-I editing and genome maintenance, particularly through factors involved in DNA repair and the DNA damage responses. To address this, we aimed to comprehensively identify inosine-modified transcripts associated with DNA repair pathways.

To explore the potential crosstalk between A-to-I RNA editing and DNA repair factor transcripts, we focused on the human lymphoblastoid cell line TK6, which is extensively used in genome stability and genotoxicity research. TK6 cells retain functional p53 and exhibit a stable karyotype, making them a reliable model for studying the relationship between RNA editing and genome maintenance. We employed the EpiPlex RNA assay (Sendinc et al., 2024), a technique that enables epitranscriptome-wide mapping of both m^6^A and inosine modifications in cellular RNAs by enriching for modifications in fragmented RNA, to perform a comprehensive analysis of RNA modification dynamics. The resulting epitranscriptomic mapping revealed significant enrichment of A-to-I editing within DNA repair-related pathways. Leveraging this dataset, we further investigated the isoform-specific roles of ADAR1 by comparing inosine modifications in DNA repair transcripts under conditions deficient in either ADAR1 p110 or p150. Notably, the nucleotide excision repair factor XPA was found to undergo splicing regulation via intronic inosines. This study provides suggests a possible link between RNA inosine modification and DNA repair processes.

## 2 Materials and methods

### 2.1 Cell culture

The human lymphoblastoid The TK6-derived human lymphoblastoid cell line TSCE5 (referred to hereafter as TK6 for simplicity) were cultured in RPMI-1640 medium (Nacalai Tesque) supplemented with 200 μg/mL sodium pyruvate, 100 U/mL penicillin, and 100 μg/mL streptomycin, and 10% (v/v) heat-inactivated fetal bovine serum (Nichirei Biosciences, Inc.) (Honma et al., 2003). The cultures were maintained at 37 °C in a 5% CO_2_ atmosphere with 100% humidity.

### 2.2 Generation of *ADAR1* deficient TK6 cell lines

To generate *ADAR1* p150 and p110 deficient cells, we designed single-guide RNA (sgRNA) targets for CRISPR/Cas9 genome editing in combination with gene targeting constructs. Primers used in the experiments are listed in Supplementary Table S1. CRISPR-target sequences are depicted in Figure 2B. sgRNAs were inserted into the *Bbs*I site of the pX330 vector. For disruption of *ADAR1* p150, the p150 targeting plasmid was constructed as follows; left and right arm fragments were PCR-amplified from TK6 genomic DNA. The amplified fragments were assembled via a seamless reaction (GeneArt^®^ Seamless Cloning and Assembly Kit; Invitrogen) into the DT-ApA/PURO^R^ vector (kindly gifted by Dr. Hiroyuki Sasanuma), which is predigested with *Apa*I and *Afl*II. The vectors pX330-gRNA/p150 (6 μg) and the p150 target plasmid (2 μg) were transfected into TK6 cells by a NEPA21 electroporator (Nepa Gene Co. Ltd.) following the manufacturer’s instructions. After 48 h incubation, cells were seeded into 96 microwell plates in the presence of puromycin (0.5 µg/mL). Drug-resistant cell colonies were picked 10–14 days after transfection and subjected to genomic PCR for a targeted allele with puromycin-resistance cassette. For disruption of *ADAR1* p110, the p110 target plasmid was constructed as follows; left and right arm fragments were PCR-amplified from TK6 genomic DNA. The DNA fragment containing the neomycin resistance marker gene conjugated with the simian virus 40 polyadenylation signal (*NEO* ^R^-polyA) was amplified from pUC-NSD2-*NEO* ^R^ (Iwasaki et al., 2024). The resulting left arm, right arm, and the *NEO* ^R^-polyA fragments were assembled via a seamless reaction (GeneArt^®^ Seamless Cloning and Assembly Kit; Invitrogen). The vectors pX330-gRNA/p110 (6 μg), and the p110 target plasmid (2 μg) were then transfected into cells by a NEPA21 electroporator (Nepa Gene Co., Ltd.). After 48 h incubation, cells were seeded into 96 microwell plates in the presence of neomycin (1.0 mg/mL). Drug-resistant cell colonies were picked 10–14 days after transfection and subjected to genomic PCR for a targeted allele with neomycin-resistance cassette of p150/p110 KO construct.

### 2.3 RT-PCR

Total RNA was extracted from cells using the NucleoSpin RNA Plus kit (Macherey-Nagel). cDNAs were synthesized from the extracted RNA with ReverTra Ace (Toyobo Co., Ltd.). The RNA was then reverse transcribed using ReverTra Ace^®^ (Toyobo, Co., Ltd.). Quantitative PCR was performed using Thunderbird^®^ Next SYBR qPCR Mix (Toyobo, Co., Ltd.) and specific primers listed in Supplementary Table S1. The expression levels of the genes were normalized to the internal *GAPDH* expression levels. To amplify the *XPA* fragments spanning exons 2–6 and exons 5–6, PCR was performed using KOD-FX-Neo (Toyobo, Co., Ltd.) and cDNA derived from the cell lines with respective primers listed in Supplementary Table S1.

### 2.4 Western blotting

Total cell extracts were fractioned on 10% SDS-polyacrylamide gels and transferred onto PVDF membranes. Membranes were blocked with 3% skim milk before incubation with primary antibodies. To detect ADAR1 and GAPDH, membranes were incubated overnight at 4 °C in Hikari A solution (Nacalai Tesque) or 3% skim milk with 1:1,000 dilution of anti-ADAR1 monoclonal antibody (D7E2M, Cell Signaling) or 1:1,000 dilution of anti-GAPDH monoclonal antibody (sc-32233, Santa Cruz), respectively. To detect XPA and γH2AX, membranes were incubated overnight at 4 °C in Hikari A solution (Nacalai Tesque) with 1:100 dilution of anti-XPA monoclonal antibody (ab2352, Abcam) or 1:1,000 dilution of anti-γH2AX monoclonal antibody (9,718, Cell Signaling). After washing with Tris-buffered saline containing 0.05% Tween 20, the membranes were incubated with a 1:4,000 dilution of anti-mouse IgG conjugated to horseradish peroxidase (Cytiva) in Hikari B solution (Nacalai Tesque) or 3% skim milk. The chemiluminescent signal were detected using Chemi-Lumi One Super or Ultra (Nacalai Tesque).

### 2.5 EpiPlex RNA assay

Epitranscriptomic profiling was conducted using AlidaBio’s EpiPlex RNA Mod Encoding kit (P/N 100108) using 2.5 µg purified RNA as input. The RNA was treated with the included DNase I according to protocol directions. Prior to mod enrichment onto EpiPlex beads, the purified RNA was mixed with spike-in controls, fragmented, end-repaired and ligated to an Illumina P7 adapter. Approximately 90% of the prepared RNA fragments were captured onto bead surfaces by mod-specific RNA binders. The remaining 10% were prepared into non-enriched solution control libraries by analogous chemistry. At the bead surfaces, captured RNA fragments are randomly primed by proximally anchored adapters containing Illumina P5 adapters, a unique molecular identifier (UMI) and an MBC (modification barcode) which was extended into cDNA by a reverse transcriptase. Sample indices were added to each sample during PCR using EpiPlex Unique Dual Index Primer Kit (P/N 224001). Libraries were sized on the Agilent TapeStation and quantified by Qubit. Separate library pools containing equal representation of each library were made for bead enriched and solution control libraries. Ribosomal RNA was depleted from each pool using Biorad SEQuoia RiboDepletion Kit (P/N 17006487) according to manufacturer’s directions. Each library was sequenced to ∼25 M reads on an Illumina NextSeq1000 using an X-LEAP SBS 200 cycle kit (P/N 20100986).

### 2.6 Peak calling

Reads were processed using EpiScout v.beta-3.3.4, a proprietary RNA modification detection pipeline developed by AlidaBio. Briefly, raw read quality was initially determined using FastQC v0.12.1. MBC and UMI barcodes were then extracted from read sequences, while low-quality bases were trimmed out. Trimmed reads with length <30 bp were removed. Clean reads were then mapped to the human genome (GRCH38.p14) using STAR v2.7.11a (Dobin et al., 2013), as well as to our internal spike-in control references using Bowtie2 v2.5.2 (Langmead and Salzberg, 2012). Genome mapped reads were split by MBC and deduplicated according to MBC and UMI barcodes using samtools v1.20 and a custom Python v3.10 script (Danecek et al., 2021).

Peak calling was performed using a custom Python script on a per-modification basis. In brief, peaks were called in a signal-processing manner using the enrichment sample as signal and solution control as a measure of background. Peak regions and fold enrichment were determined using the Python package scipy find_peaks and scaled according to the exogenous spike-in controls’ enrichment factor resulting in final peak fold-enrichment (Virtanen et al., 2020). To identify high-confidence peaks, EpiScout employed an ensemble classification algorithm including hidden Markov and random forest models to determine and filter peaks based on model log probabilities. These probabilities are reported as PHRED scale q-scores. Peaks which passed this cutoff, along with a minimum depth requirement of 5 reads in the enrichment sample, were considered to be “high-confidence”. After filtering, peaks were annotated using the GRCh38.p14 GTF and fasta files using a custom Python script. Differential analysis was performed using bedtools and a custom Python script. Gene clustering of peaks was performed using the Python package sklearn AgglomerativeClustering (Pedregosa et al., 2011). Aggregated z-scores were then clustered using Euclidean affinity and ‘ward’ linkage algorithms.

### 2.7 Variant calling

To accurately identify A-to-G variants captured within EpiPlex inosine peak calls, we used the BCFtools commands mpileup, norm, call, and filter with the mpileup parameters (--full-BAQ, --max-depth 20000), call parameters (-mA), and filter criteria (min (FMT/DP) > 5)) (Pedregosa et al., 2011). Only sites which overlapped inosine peak calls and had a variant rate ≥10% were reported.

### 2.8 Motif analysis

Motif discovery using MEME was performed to identify enriched sequence motifs within inosine peak regions detected in DNA repair genes. The default parameters were used, except that the number of motifs to find was set to 10. To assess whether the identified motifs correspond to known repetitive elements, homology searches were conducted against the Dfam database using *Homo sapiens* as the reference organism.

### 2.9 RNA sequencing and data analysis of gene expression profiles

Total RNA was extracted from 2 × 10^6^ cells using the NucleoSpin RNA Plus Kit (Macherey-Nagel, Düren, Germany) following the manufacturer’s instructions. RNA-seq libraries were prepared using the TruSeq Stranded mRNA LT Sample Prep Kit (Illumina, San Diego, CA) according to the manufacturer’s protocol. Sequencing was conducted on the NovaSeq X Plus in 100 bp Paired End mode, using the NovaSeq X series 10B reagent Kit. The raw RNA-seq data underwent adapter trimming and low-quality region removal with Trimmomatic 0.38. The resulting trimmed reads were aligned to the reference genome (GRCh38/hg38) using HISAT2, with transcript assembly conducted using StringTie. Differential expression analysis was carried out with edgeR (v.4.2.2), using raw gene-level read counts. Genes with low expression were excluded by filtering for CPM≧10 across the libraries. After filtering, normalization was conducted using the trimmed mean of M values (TMM) method. Differentially expressed genes (DEGs) were identified using glmQLFTest. Genes with p-value <0.05 and log2 fold change >1 were considered significantly DEGs. Gene Ontology (GO) analysis of upregulated and downregulated DEGs was performed using DAVID.

### 2.10 Gene ontology and pathway enrichment analysis

Gene ontology enrichment analysis was performed using DAVID, focusing on the Biological Process (GO:BP) category. *Homo sapiens* was selected as the organism, identifiers were provided as OFFICIAL_GENE_SYMBOL. Enriched GO terms with a p-value < 0.05 were considered statistically significant.

### 2.11 UV irradiation

Cells were washed and resuspended with RPMI-1640 without phenol red prior to irradiation. Cells (2.5 × 10^6^) in 5 mL medium were exposed to ultraviolet C (UVC) light (0, 1, 2, 3, and 4 J/m^2^) in 10 cm Petri dishes. Irradiated cells were then seeded into 96-microwell plates at 8 cells/mL (1.6 cells/well) to determine cell survival. Colonies in 96-microwell plates were scored after 14–21 days.

### 2.12 Chemical treatment

Cisplatin and camptothecin (CPT) were purchased from FUJIFILM Wako Pure Chemical Corporation (Osaka, Japan). Cisplatin was dissolved in 0.9% NaCl solution. CPT was dissolved and diluted in dimethyl sulfoxide. For cellular exposure to the chemicals, 20 mL of cell suspensions at a concentration of 5 × 10^6^ cells/mL was treated with CPT (0, 0.5, 1, 3, and 5 nM) or cisplatin (0, 0.25, 0.5, 1, and 1.5 µM). After 24 h of treatment at 37 °C, cells were washed with RPMI1640 medium twice and then seeded into 96-microwell plates to determine cell survival. Colonies in 96 microwell plates were scored after 14–21 days.

## 3 Results

### 3.1 Global profiling of A-to-I RNA editing in human TK6 cells

**FIGURE 1:**
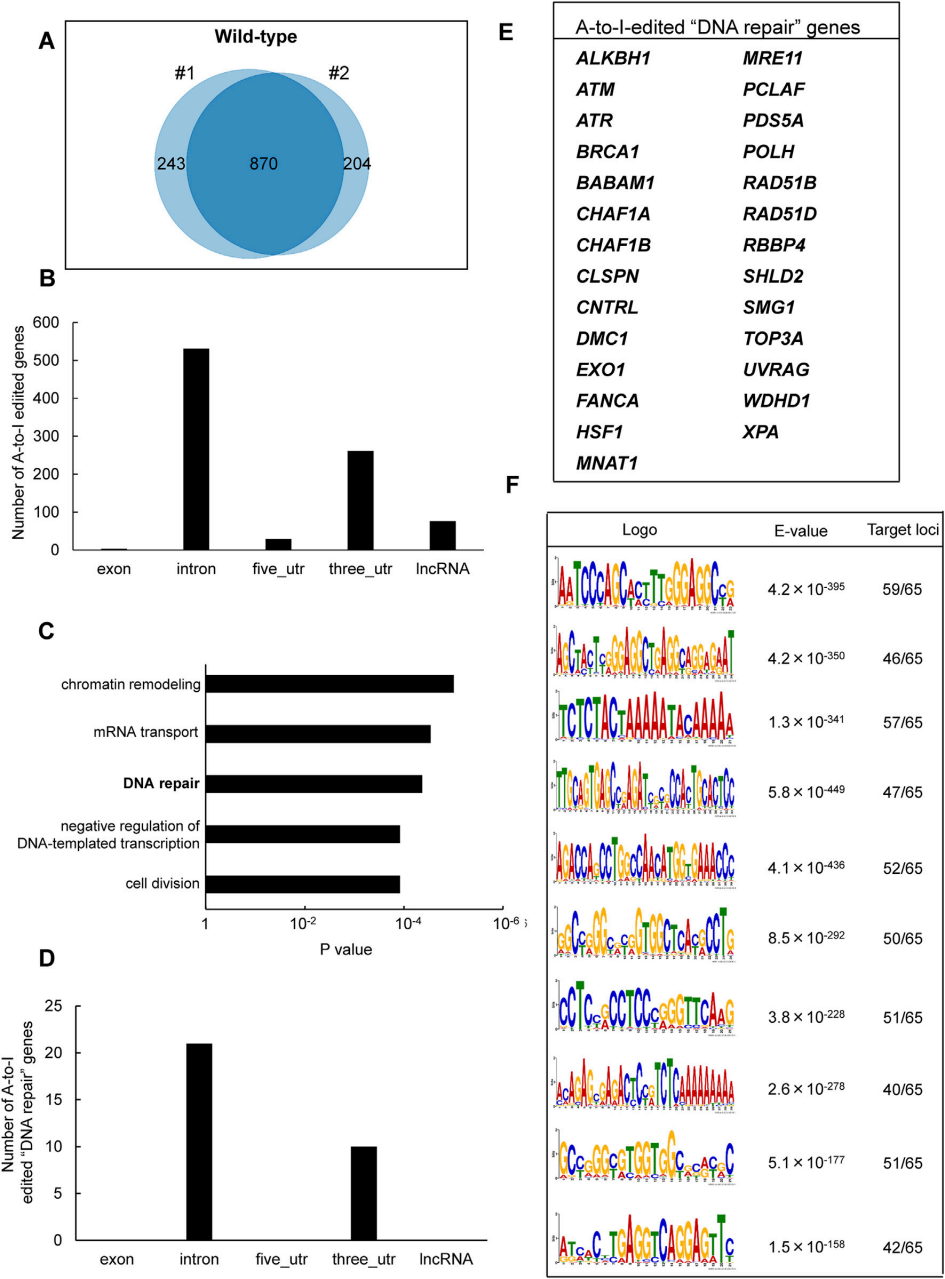
Identification of A-to-I RNA editing sites and associated biological pathways in human TK6 cells. **(A)** Venn diagram showing inosine-modified genes detected by the EpiPlex assay in two biological replicates (#1 and #2) of wild-type TK6 cells. **(B)** Distribution of A-to-I editing peaks in transcripts expressed in wild-type TK6 cells. Peaks commonly detected across two independent biological replicates are shown. **(C)** GO enrichment analysis of genes encoding A-to-I-edited RNAs. Statistical significance was determined at p < 0.05, and the top five enriched biological processes are shown. **(D)** Distribution of A-to-I editing peaks within transcripts of genes involved in DNA repair. **(E)** List of genes classified under the DNA repair category based on GO analysis. **(F)** Top ten enriched sequence motifs in A-to-I–edited transcripts of DNA repair-associated genes (MEME analysis). E-values indicate statistical significance. Target loci represent 65 inosine peaks from 27 DNA repair genes.

To identify inosine-modified transcripts in human lymphoblastoid TK6 cells, two independent EpiPlex assays were performed using wild-type (WT) cells. The assays detected 2,308 and 2,251 inosine peaks in WT#1 and WT#2, respectively (Table 1; Supplementary Table S2). Notably, the median A-to-I density within EpiPlex peaks was approximately 7–8 sites per 100 bp in TK6 cells (Supplementary Figures S1A,B). REDIportal-annotated A-to-I sites showed a comparable density, with median values around 15–20 per 100 bp (Supplementary Figure S1C). A total of 870 genes were commonly identified in both datasets (Figure 1A), demonstrating consistent detection of inosine modifications across biological replicates. Among these 870 genes, A-to-I–modifications were distributed across exons (3 genes), introns (531 genes), 5′-untranslated regions (5′UTRs; 29 genes), 3′UTRs (261 genes), and long non-coding RNAs (lncRNAs; 76 genes) (Figure 1B). These figures include transcripts with modifications detected in multiple regions.

**TABLE 1:** Number of inosine- and m^6^A-peaks detected in human TK6 cells by EpiPlex assay.

Cell line	Number of peaks containing inosine	Number of peaks containing m^6^A
WT#1	2308	11040
WT#2	2251	10786
p150 KO#1	736	11827
p150 KO#2	479	9771
p150/p110 KO#1	4	10884
p150/p110 KO#2	2	11282

To explore the biological role of the genes containing A-to-I-modified RNAs, GO enrichment analysis was conducted using the DAVID bioinformatics tool. The analysis revealed significant enrichment of genes involved in “chromatin remodeling,” followed by “mRNA transport,” “DNA repair,” “negative regulation of DNA-templated transcription,” and “cell division” pathways (Figure 1C). The genes associated with each pathway are listed in Supplementary Table S3. We focused on DNA repair-related genes because the maintenance of genome integrity is central to this study and ADAR1 has been implicated in DNA damage responses. Based on the GO results, 27 genes were classified under DNA repair-related pathways (Figure 1D). These included factors involved in DNA double-strand break (DSB) repair (i.e., *ATM, ATR, BRCA1, MRE11, TOP3A, EXO1, RAD51B, RAD51D, SHLD2*), nucleotide excision repair (i.e., *XPA*), mismatch repair (i.e., *EXO1*), base excision repair (i.e., *ALKBH1*), interstrand crosslink repair (e.g., *FANCA*), and translesion DNA synthesis (i.e., *POLH*) (Figure 1E). Among these 27 genes, A-to-I–modifications were located in introns (21 genes) and 3′UTRs (10 genes). The inosine peaks were obtained with high reproducibility across two biological replicates (Supplementary Figure S2). To further examine the sequence characteristics of A-to-I–edited sites, motif analysis was performed using MEME (Multiple EM for Motif Elicitation) (Bailey and Elkan, 1994), on 65 inosine peak sequences from the 27 DNA repair-related genes. As shown in Figure 1F, shared sequence motifs were identified across several peak sequences. To determine whether these motifs matched known repetitive elements, homology searches were conducted using the Dfam database. As a result, motif 2 correspond to AluYh7, motif 4 to AluYc, and motif 5 to AluSq4. The majority of A-to-I sites in representative DNA repair genes (*ATR, POLH, ATM,* and *FANCA*) were located within Alu elements (>90%) (Supplementary Figure S1D).

### 3.2 Differential contribution of ADAR1 isoforms to A-to-I RNA editing

**FIGURE 2:**
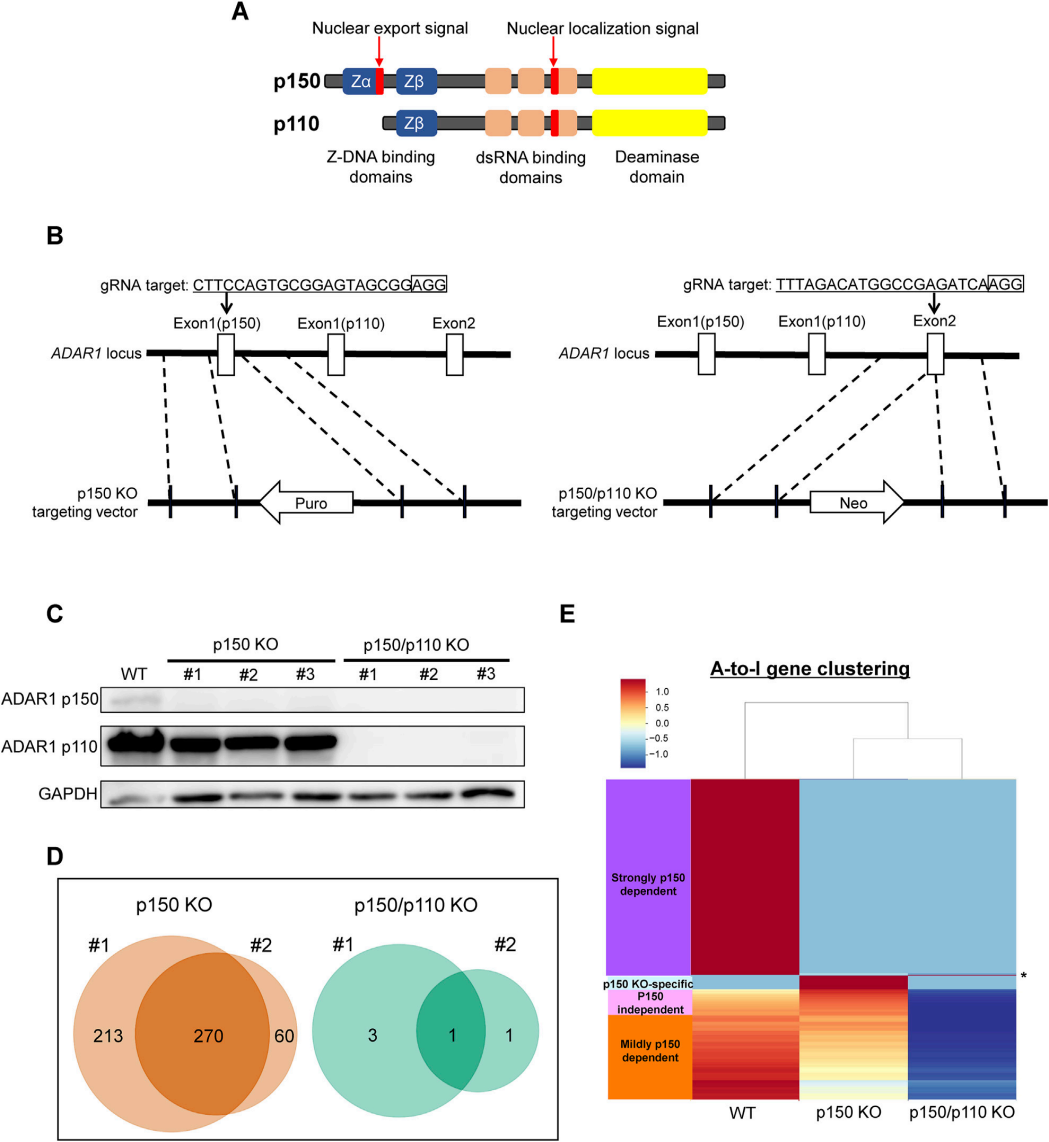
Generation of ADAR1-deficient cell lines for isoform-specific A-to-I RNA editing analysis. **(A)** Domain structure of the two ADAR1 isoforms, p150 and p110. **(B)** Schematic representation of the CRISPR/Cas9-mediated disruption of ADAR1 p150 and p150/p110. The target sequence and corresponding vectors containing either a reverse-oriented puromycin- or neomycin-resistance cassette are illustrated. **(C)** Western blot analysis of ADAR1 isoforms. Whole-cell extracts from WT, p150 KO clones (#1, #2, #3), and p150/p110 KO clones (#1, #2, #3) were resolved by 10% SDS-PAGE. GAPDH was used as a loading control. **(D)** Venn diagrams showing inosine-modified genes detected by the EpiPlex assay in two biological replicates (#1 and #2) of p150 KO (left) and p150/p110 KO (right) TK6 cells. **(E)** Clustering analysis of genes exhibiting A-to-I editing. Based on inosine peak scores (fold-enrichment ratio) in WT and p150 KO cells, genes were categorized as strongly p150 dependent (WT/(p150KO+1) ≧4; purple), mildly p150 dependent (1<WT/(p150KO+1) <4; orange), p150 independent (WT/(p150KO+1) <1; pink), and newly detected upon p150 loss, i.e., p150KO-specific (WT = 0, p150KO > 1, cyan). Asterisk (*) indicates the genes categorized as p150/p110 KO-specific (WT = 0, p150KO = 0, p150/p110KO > 1).

To assess isoform-specific contributions to inosine modifications, three independent ADAR1 p150-deficient (p150 KO) TK6 cell clones were generated (Figures 2A,B). These p150 KO cells allowed for distinction between editing mediated by p150 specifically and total ADAR1 activity. Notably, the expression of ADAR1 p110 remained largely unchanged in p150 KO cells (Figure 2C). To further delineate isoform contributions, ADAR1 p150/p110 deficient (p150/p110 KO) cells were established by disrupting p110 expression in the p150 KO background (Figures 2B,C). Comprehensively profiling using the EpiPlex assay was conducted on two independent clones each from WT, p150 KO, and p150/p110 KO cells. Inosine peak counts were markedly reduced in the p150 KO#1 and p150 KO#2 clones to 736 and 479, respectively, compared to 2,308 and 2,251 in WT#1 and WT#2 (Table 1; Supplementary Table S2). In contrast, only 4 and 2 peaks were detected in p150/p110 KO#1 and p150/p110 KO#2, respectively, indicating a 73.4% reduction in p150 KO cells and a 99.9% reduction in p150/p110 relative to WT (Supplementary Figures S1A–C). At the gene level, 270 inosine-modified genes were commonly observed across both p150 KO clones, whereas only a single gene was shared between the two p150/p110 KO clones (Figure 2D). Notably, m6A peak counts did not significantly differ across WT and KO lines (Table 1). To capture a broader landscape of A-to-I editing and determine p150-dependency, clustering analysis was performed on transcripts containing editing sites in at least one WT sample (Supplementary Table S2). As shown in Figure 2E and Supplementary Table S4, genes were categorized based on inosine peak scores (fold-enrichment ratio) in WT and p150 KO cells as follows: strongly p150 dependent (WT/(p150KO+1) ≥ 4; purple), mildly p150 dependent (1 < WT/(p150KO+1) < 4; orange), p150 independent (WT/(p150KO+1) < 1; pink), and p150KO-specific (WT = 0, p150KO > 1; cyan). Clustering categorized 839 genes as strongly p150-dependent, 303 genes as moderately p150-dependent (reduced in p150 KO), and 176 genes as p150-independent (similar or increased in p150 KO). Additionally, 58 genes exhibited new inosine peaks in p150 KO cells, suggesting a complex regulatory landscape.

**FIGURE 3:**
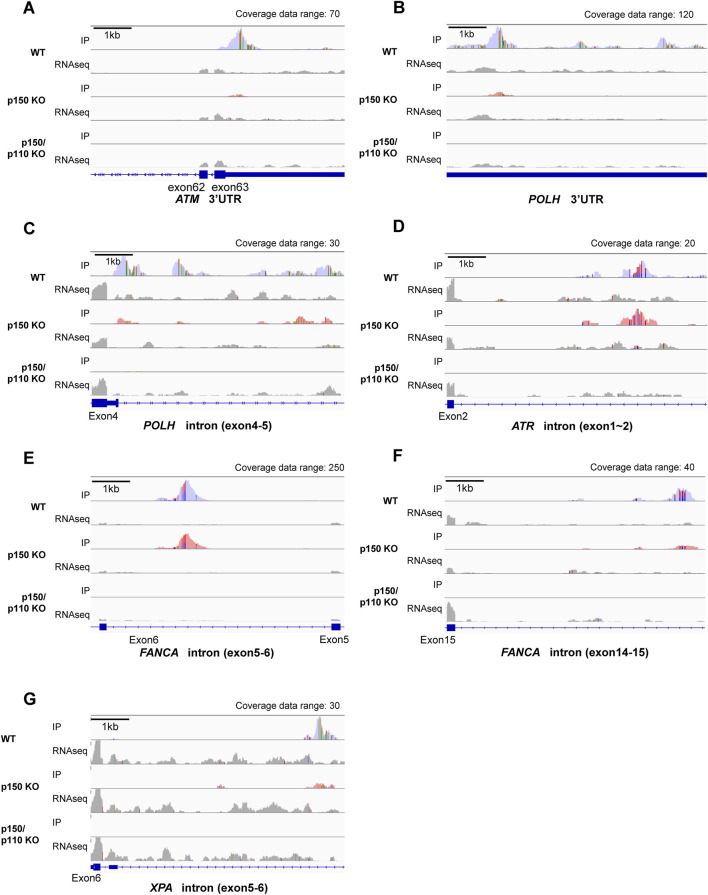
Visualization of A-to-I RNA editing loci in transcripts of DNA repair-related genes in wild-type and ADAR1-deficient TK6 cells. Read coverage profiles are shown for representative DNA repair-associated genes: **(A)** *ATM* (3′UTR), **(B)** *POLH* (3′UTR), **(C)** *POLH* (intron between exon4-5), **(D)** *ATR* (intron between exon1-2), **(E)** *FANCA* (intron between exon5-6), **(F)**, *FANCA* (intron between exon14-15), and **(G)** *XPA* (intron between exon5-6) in wild-type (blue), p150 KO (pink), and p150/p110 KO cells (green). The y-axis represents read counts and the x-axis indicates genomic coordinates. The scale bar corresponds to 1 kb. “Coverage data range” in the top-right corner of each panel indicates the maximum coverage value. IP tracks indicate inosine peak calls, while gray peaks labeled “RNAseq” represent input RNA sequencing data. Colored regions within coverage tracks highlight A-to-I RNA editing sites. Strand-specific colors compositions are shown: green (Adenosine) and orange (Guanosine) for forward reads; red (Thymidine) and blue (Cytidine) for reverse reads, facilitating visual estimation of nucleotide ratios at individual position.

Among the 27 DNA repair-related genes with A-to-I-editing, 17 were strongly p150-dependent, 8 were moderately p150-dependent, and 2 were p150-independent (Table 2). To further examine the abundance and distribution of editing sites, transcript visualization using Integrative Genomics Viewer (IGV) was performed. In the 3′UTR, 22 edited bases were found in *ATM* (Figure 3A; Supplementary Table S5), and 78 in *POLH* (Figure 3B; Supplementary Table S5). Within introns, 122 edited bases were detected in *POLH* (Figure 3C; Supplementary Table S5), 22 in *ATR* (Figure 3D; Supplementary Table S5), 36 in *FANCA* (Figures 3E,F; Supplementary Table S5), and 19 in *XPA* (Figure 3G; Supplementary Table S5). Thus, the extent of editing varied markedly among genes.

**TABLE 2:** ADAR1 isoform dependency of A-to-I editing in transcripts of DNA repair genes.

Isoform dependency	Locus of A-to-I editing				
	Intron			Three_UTR	
Strongly p150 dependent	*ALKBH1*	*MNAT1*	*UVRAG*	*ATM*	*RBBP4*
	*BABAM1*	*PCLAF*	*WDHD1*	*HSF1*	
	*CHAF1A*	*RBBP4*	*XPA*	*MRE11*	
	*EXO1*	*SHLD2*		*PCLAF*	
	*HSF1*	*SMG1*		*TOP3A*	
Mildly p150 dependent	*ATR*	*PDS5A*		*CHAF1B*	
	*BRCA1*	*POLH*		*CLSPN*	
	*DMC1*	*RAD51B*		*POLH*	
p150 independent	*CNTRL*			*RAD51D*	
	*FANCA*				

### 3.3 ADAR1 deficiency affects *ATM* expression and *XPA* splicing

**FIGURE 4:**
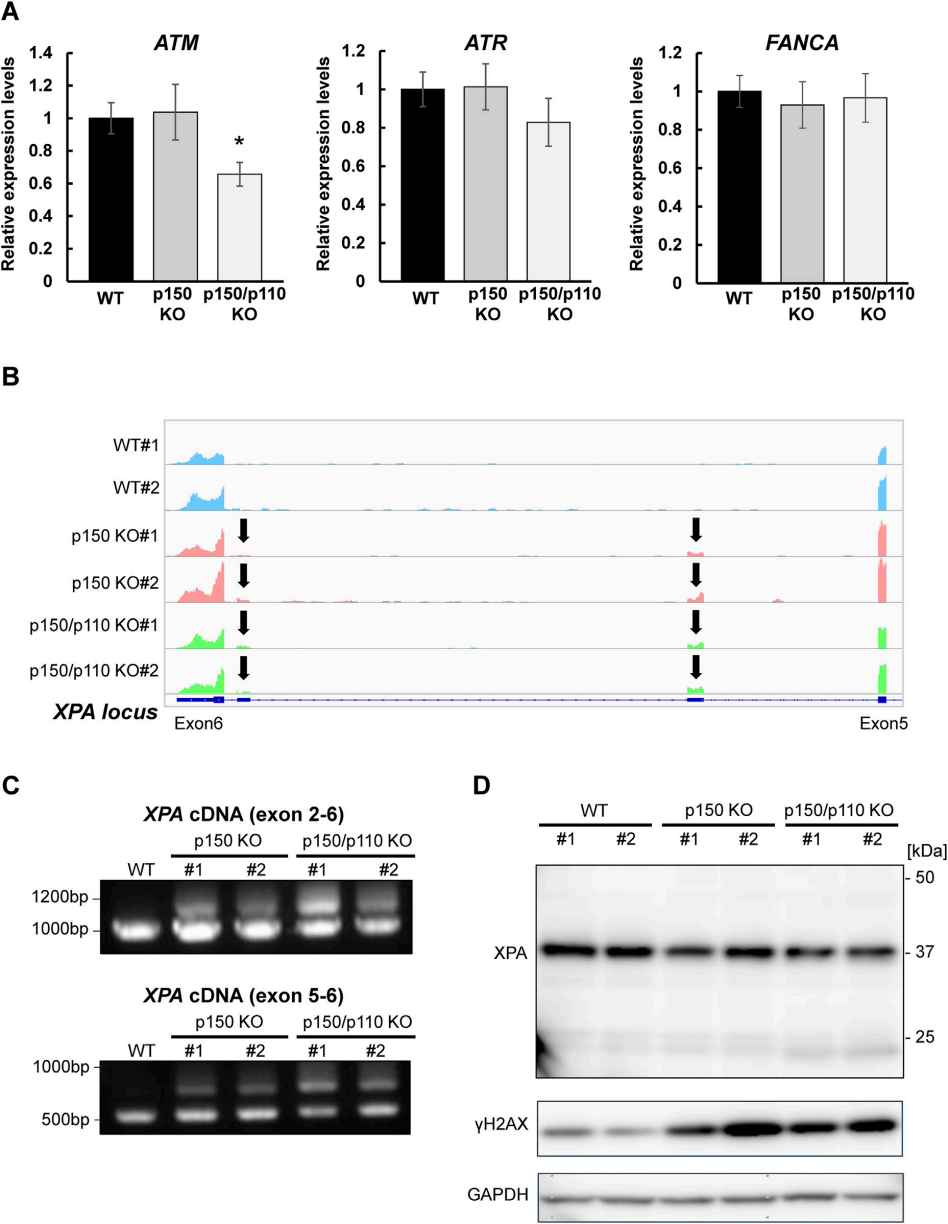
Effects of ADAR1 deficiency on *ATM* expression and *XPA* splicing. **(A)** Gene expression levels of *ATM, ATR,* and *FANCA* were quantified by RT-qPCR in WT (black), p150 KO (gray), and p150/p110 KO (white) cells. Data represent six independent experiments (n = 6). Statistical significance was determined by Student’s t-test (p < 0.05). **(B)** RNA-seq read coverage profiles of the *XPA* gene in WT#1, WT#2, p150 KO#1, p150 KO#2, p150/p110 KO#1, and p150/p110 KO#2 cells. Arrows indicate novel splicing peaks between exon 5 and 6 of the main splicing variant. **(C)** Detection of *XPA* splicing variants. PCR was performed using cDNA from WT#1, p150 KO#1, p150 KO#2, p150/p110 KO#1, and p150/p110 KO#2, with primers targeting exons 2–6 and 5–6. Amplified products were resolved on 1.0% agarose gels. For amplification between exons 2–6, expected band sizes were 946 bp for variant 1 and 1,260 bp for a potential alternative variant. For amplification between exons 5–6, expected sizes were 544 bp for variant 1 and 964 bp for alternative variants. **(D)** Western blot analysis for XPA and γH2AX protein. Whole cell extracts from WT#1, WT#2, p150 KO#1, p150 KO#2, p150/p110 KO#1, and p150/p110 KO#2 cells were loaded onto a 5%–20% gradient SDS-polyacrylamide gel. GAPDH served as the internal control.

To evaluate how A-to-I–modifications influence DNA repair gene transcripts, *ATM* (3′UTR modification), *ATR*, and *FANCA* (intronic modifications) were selected, as these genes constitute central components of DNA damage response (DDR) pathways and several of them have been frequently reported in the context of ADAR1-related editing. As shown in Supplementary Figure S3, RNA-seq analyses revealed upregulation of interferon-stimulated genes in both p150 KO and p150/p110 KO cells compared with WT cells, but no significant enrichment of downregulated genes in the DNA repair pathway. RNA expression levels were measured via qPCR in WT, p150 KO, and p150/p110 KO cells. As shown in Figure 4A, *ATM* expression significantly decreased in p150/p110 KO cells compared to WT, in agreement with a previous study (Sagredo et al., 2018), while p150 KO cells showed no significant difference. In contrast, *ATR* and *FANCA* expression levels remained consistent across all three cell lines. Splicing patterns of intron-modified transcripts were also analyzed using IGV analysis of RNA-seq data. We surveyed all genes categorized under DNA repair pathways for potential splicing alterations, and identified XPA as the only gene showing an altered splicing pattern associated with ADAR1 deficiency. In the *XPA* gene, two novel peaks emerged between exons 5 and 6 in both p150 KO and p150/p110 KO cells compared to WT (Figure 4B). To validate these splicing events, RT-PCR followed by agarose gel electrophoresis was performed. Additional cDNA bands appeared in the KO cells relative to WT, indicating that ADAR1-mediated A-to-I editing influences *XPA* mRNA splicing (Figure 4C). Western blot analysis revealed that XPA protein was expressed at physiologically relevant levels across all cell lines (Figure 4D). In contrast, the DDR marker γH2AX levels were markedly elevated in p150 KO and p150/p110 KO cells.

### 3.4 Effects of ADAR1 deficiency on cellular sensitivity to DNA-damaging agents

**FIGURE 5:**
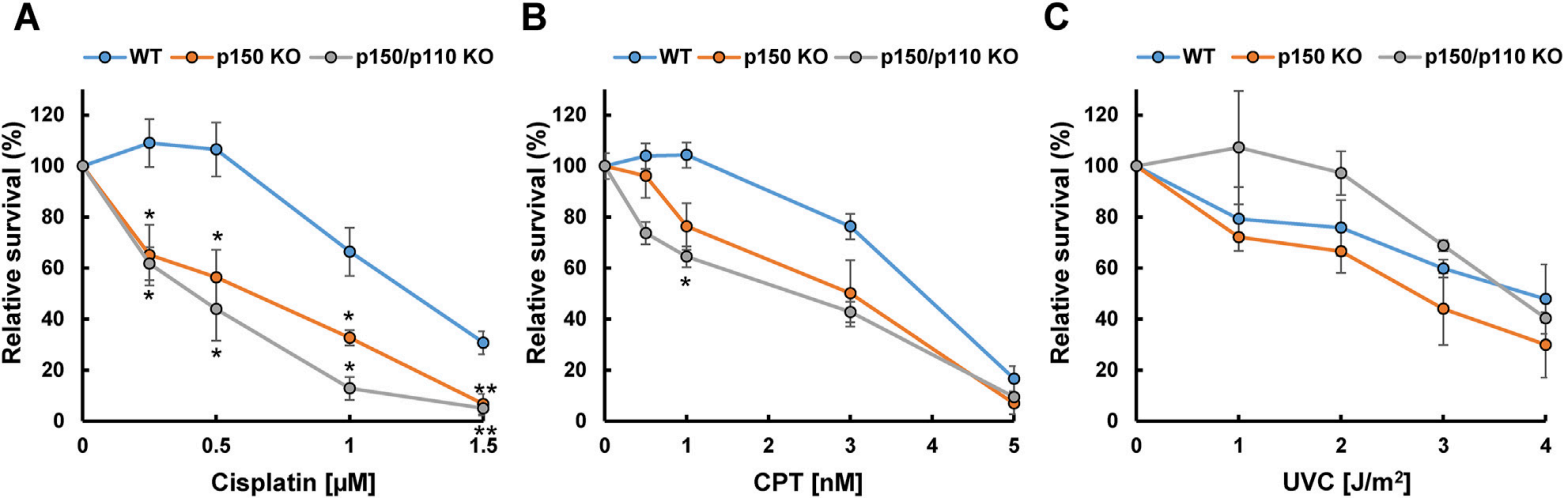
Cytotoxicity of DNA-damaging agents in ADAR1-deficient human TK6 cells. Relative cell survival (%) of WT (blue), p150 KO (orange), and p150/p110 KO (grey) cells following treatment with cisplatin **(A)**, CPT **(B)**, and UVC **(C)**. Values presented are means ± SEM of 2–6 independent experiments. Statistical analysis was performed using Dunnett’s test; p < 0.05, p < 0.01.

To examine the functional role of ADAR1 in the tolerance to genotoxic stress, survival rates of WT, p150 KO, and p150/p110 KO cells were evaluated after treatment with DNA-damaging agents, including cisplatin, CPT, and UVC. Treatment with cisplatin (0.25–1.5 µM) resulted in significantly increased sensitivity in both p150 KO and p150/p110 KO cells across all tested concentrations compared to WT (Figure 5A). Among the CPT concentrations tested, p150/p110 KO cells showed significantly increased sensitivity compared to WT cells at 1 nM CPT (Figure 5B). Disruption of the p150 isoform alone also slightly sensitized cells to CPT, although the difference was not statistically significant. In contrast, UVC exposure did not produce significant survival differences among any of the cell lines (Figure 5C). These findings suggest that ADAR1 deficiency exerts variable effects depending on the type of genotoxic stress and the nature of DNA damages. In particular, abnormal *XPA* splicing observed in p150 KO and p150/p110 KO cells may influence cellular functions in ways not directly linked to UV sensitivity.

## 4 Discussion

Recent studies have revealed that A-to-I RNA editing plays a regulatory role in DNA repair mechanisms. For example, in the human breast carcinoma cell line ZR-75-1, A-to-I editing of *ATM* and *POLH* transcripts is enhanced, accompanied by elevated expression levels of these genes, as shown by RNA-seq analysis (Sagredo et al., 2018). In human U2OS cells, depletion of ADAR2 has been reported to increase γH2AX levels and the frequency of micronucleus formation, indicating the accumulation of DNA damage (Jimeno et al., 2021). Moreover, ADAR1-mediated A-to-I editing influences cellular responses to DNA-damaging agents: knockdown of ADAR1 significantly reduces cell viability following cisplatin treatment in cholangiocarcinoma HuCCT1 and RBE cell lines (Liu et al., 2024). This phenotype can be rescued by reintroducing wild-type ADAR1, but not by a catalytically inactive mutant (ADAR1 E912A), suggesting that A-to-I RNA editing activity is essential for cisplatin resistance. Thus, A-to-I modification likely plays a critical role in genome stability, and elucidating its broader functions may contribute to novel therapeutic strategies in cancer. Therefore, this study’s analysis of the relationship between A-to-I RNA editing and DNA repair pathways provides insight that may help to understand the physiological relevance of inosine modifications in genome maintenance.

In this study, the EpiPlex assay, a recently developed for epitranscriptome analysis, was applied to the human lymphoblastoid TK6 cell line, identifying 870 A-to-I-modified transcripts across two independent biological replicates (Supplementary Table S2). WT cells contained on average 68,801 A-to-I editing sites. EpiPlex peaks called in WT cells were also validated using the REDIportal v3 database (http://srv00.recas.ba.infn.it/atlas/) (Picardi et al., 2017), containing on average 156,556 sites reported by the database (Supplementary Figure S1A). A-to-I density within EpiPlex peaks showed a wide distribution in WT and p150 KO cells, with a right-skewed tail representing peaks of particularly high A-to-I density, indicative of hyper-editing regions (Supplementary Figures S1B,C). The majority of editing sites were located in intronic regions, followed by 3′UTRs (Figure 1B). This distribution pattern aligns with the global annotation of RNA editing sites annotated in the RADAR database of human transcripts (Weirick et al., 2019; Ramaswami and Li, 2014). According to GO enrichment analysis, these edited transcripts were significantly enriched in pathways such as “chromatin remodeling” and “DNA repair” (Figure 1C; Supplementary Table S3). Notably, associations between specific biological pathways and RNA modifications appear to be cell type-dependent. For example, in brain tissues, A-to-I editing targets are enriched in transcriptional regulation and neurological disorder–related pathways (Sakurai et al., 2014), while in granulosa cells from patients with polycystic ovary syndrome, editing events are associated with the mitotic cell cycle and membrane transport (Kong et al., 2023). In 14 human cell lines from the ENCODE project, modified RNAs are enriched in cell division, antiviral response, and translational control pathways (Park et al., 2012). Collectively, these findings indicate that the functional significance of A-to-I RNA editing varies by tissues and cell type. Our analysis using TK6 cells revealed that the presence of Alu elements within DNA repair–related loci serves as a major target of A-to-I editing, consistent with previous observations that repetitive elements frequently harbor editing sites.

Notably, we identified A-to-I–modifications in mRNAs of 27 DNA repair-related genes, including previously reported targets *ATM* and *POLH* (Figure 1E) (Sagredo et al., 2018). Of modified sites identified in these targets, >95% were annotated as Alu elements by REDIportal (Supplementary Figure S1D). Furthermore, the A-to-I peak sequences of these transcripts shared conserved sequence motifs (Figure 1F), some of which showed homology to Alu subfamily elements such as AluYh7, AluYc, and AluSq4. This is consistent with earlier reports that A-to-I editing preferentially occurs within Alu elements (Sawyer et al., 2004; Godoy et al., 2012; Nishikura, 2016), suggesting that A-to-I editing in DNA repair transcripts relies on double-stranded RNA structures formed by Alu-derived sequences. In addition to the DNA repair pathway, GO analysis indicated enrichment in “chromatin remodeling” (Figure 1C), including genes encoding topoisomerase I (*TOP1*) and histone-modifying enzymes, (*KDM2A, KDM4B, NSD1*, and *NSD2*). These enzymes are known to contribute to the regulation of DNA damage responses (Iwasaki et al., 2024; Pommier et al., 2006; Khoury-Haddad et al., 2015; Wagner et al., 2025), implying that A-to-I editing may support genome integrity through broader chromatin-based repair mechanisms.

Using the EpiPlex assay to compare wild-type, p150 KO, and p150/p110 KO cell lines, we assessed isoform-specific contributions of ADAR1 to A-to-I editing. Inosine peak levels were reduced by approximately 73.4% in p150 KO cells and by 99.9% in p150/p110 KO cells relative to wild-type cells. We note that p150 uniquely harbors a Zα binding domain (Figure 2A), which enables recognition of Z-DNA and Z-RNA and contributes to its broader editing activity compared with p110. Previous reports have demonstrated that this domain of p150 plays an important role in A-to-I editing activity and accounts for the majority of editing events in cells (Nakahama et al., 2021), which may explain the predominant contribution of p150 to RNA editing observed in our study. In contrast, the number of m6A peaks remained unchanged, suggesting that A-to-I and m6A modifications are independent processes (Figure 3A). Cluster analysis of A-to-I editing revealed 839 genes as strongly p150-dependent, 303 as moderately p150-dependent, and 176 as p150-independent. Additionally, a distinct subset of genes was edited specifically by the p110 isoform (Figure 3B). This contrasts with a previous report in HEK293 cells where no p110-specific editing was observed (Sun et al., 2021), suggesting that ADAR1 editing targets vary by cell type and reflect isoform-specific localization and activity. Consistently, A-to-I editing has been shown to occur in a cell type–specific manner in human brain cells (Cuddleston et al., 2022). Moreover, under stress conditions, p110 undergoes phosphorylation, promoting transient cytoplasmic translocation (Sakurai et al., 2017). Among the DNA repair factors identified in this study, isoform dependency appeared to vary across individual genes, implying potential gene-specific contributions of p150 and p110 to A-to-I editing.

Regarding the effects of ADAR1 deficiency on cell viability, our study demonstrated that TK6 cells lacking ADAR1 remain viable, in contrast to previous reports where ADAR1 loss induced cell death in HeLa cells. In ALT (alternative lengthening of telomeres)-negative cells like HeLa, ADAR1p110 is required for telomerase-dependent telomere maintenance by editing RNA:DNA hybrids and resolving telomeric R-loops (Shiromoto et al., 2021): Loss of ADAR1 in these cells destabilizes telomeres, leading to lethal proliferative arrest. In contrast, ALT-positive cells, characterized by heterogeneous telomere lengths, maintain telomere through telomerase-independent mechanisms (Loe et al., 2020). The viable phenotype of TK6 cells lacking ADAR1 may suggest the presence of alternative telomere maintenance pathways compensating for the loss of inosine-mediated stability. Supporting this idea, a previous study reported that telomerase does not contribute to telomere elongation following ionizing radiation in TK6 cells (Berardinelli et al., 2012).

Gene expression profiling of ADAR1-deficient cells revealed upregulation of interferon-stimulated genes in both p150 KO and p150/p110 KO cells compared to WT cells (Supplementary Figure S3), consistent with studies showing that the loss of A-to-I editing leads to accumulation of endogenous double-stranded RNAs and activation of type I interferon responses (Chung et al., 2018; Kuster et al., 2015; Bardon et al., 2016). RT-qPCR analysis revealed that *ATM* mRNA levels were downregulated in p150/p110 KO cells (Figure 4A), consistent with previous findings that ADAR1 knockdown breast cancer cells reduces *ATM* expression without affecting mRNA stability in ZR-75-1 (Sagredo et al., 2018). In contrast, expression levels of *ATR* and *FANCA* remained unchanged (Figure 4A), indicating gene and locus-specific effects of A-to-I editing on transcript expression. It should be noted that no DNA repair-related genes shown in Figure 1D were detected as DEGs in RNA-seq, indicating that the limited sensitivity of the RNA-seq analysis in quantifying subtle changes in the transcript abundance. It is also plausible that A-to-I editing affects mRNA stability. More specialized approaches, such as RNA metabolic labeling or transcriptome-wide decay profiling, for example, RATE-seq (Neymotin et al., 2014), will be required in future studies to determine whether and how A-to-I editing affects transcript stability. Notably, our RNA-seq analysis revealed that PARP1, a DNA repair factor, was upregulated in ADAR1-deficient cells (Supplementary Figure S3A). Since PARP1 was not identified as an inosine-modified transcript in our dataset, this upregulation is unlikely to be a direct consequence of A-to-I editing, but rather represents an indirect effect mediated by DDR-associated stress responses (Pathikonda et al., 2020).

Interestingly, increased expression of novel splicing variants was observed in both p150 KO and p150/p110 KO cells relative to WT (Figures 4B,C). These alternative splicing events correspond to the pathogenic non-coding transcript variants NR_149093.2 and NR_149094.2 (Tanioka et al., 2005). It is plausible that A-to-I editing within central intronic region of *XPA* alters splicing factors binding specificity, thereby reshaping splicing patterns and promoting the production of alternative variants. Supporting this possibility, intronic A-to-I editing has been implicated in splicing regulation at other gene loci. For example, SRSF1 binding to an A-to-I site promotes exonization in the *HNRPLL* gene (Chen et al., 2018), while SRSF7 binding enhances exon inclusion in *CDDC15* (Tang et al., 2020). In the *RELL2* gene, ADAR2 binds to a double-stranded RNA structure near the exon, interfering with U2AF65 binding and inducing exon skipping (Tang et al., 2020). These findings raise the possibility that ADAR1 directly regulates splicing patterns in *XPA* through similar mechanisms. At the protein level, notably, the intact XPA protein was expressed physiologically relevant levels in WT, p150, and p150/p110 cells (Figure 4D). This may implicate that the alternative splicing variant is not efficiently translated into detectable protein products and could be subject to post-transcriptional degradation, such as through nonsense-mediated mRNA decay (Lykke-Andersen and Jensen, 2015). On the other hand, markedly elevated levels of γH2AX were detected in p150 KO and p150/p110 KO cells compared to WT cells (Figure 4D), indicating altered DDR signaling linked to the absence of ADAR1 function.

To further dissect the DNA damage-specific phenotypes caused by ADAR1 deficiency, we assessed cell survival following exposure to different DNA-damaging agents. Strikingly, both p150 KO and p150/p110 KO cells exhibited a clear reduction in cell survival compared with WT cells after cisplatin treatment (Figure 5A). In contrast, sensitivity to CPT was limited: p150/p110 KO cells showed a significant decrease only at a single dose (1 nM), while p150 KO cells exhibited a modest but statistically not significant decrease compared to WT cells (Figure 5B). Interestingly, ADAR1-deficient cells exhibited no reduction in survival relative to wild-type cells after UVC irradiation (Figure 5C). These distinct patterns of sensitivity likely reflect the types of DNA lesions induced by each agent. Cisplatin generates mono-adducts and intra-strand crosslinks, which are repaired mainly by nucleotide excision repair and related pathways, and to a lesser extent interstrand crosslinks that are processed by the interstrand crosslink repair machinery (Ahmed et al., 2024). The accumulation of such lesions is expected to eventually give rise to DSBs, requiring DSB repair pathways. CPT, on the other hand, induces DNA breaks specifically during DNA replication via formation of DNA-protein crosslinks. UVC irradiation generates UV-induced dimers, which are repaired by nucleotide excision repair. Notably, a key factor in the UV tolerance is translesion DNA synthesis by Pol η, which can bypass UV-induced dimers with remarkable efficiency and high fidelity (Masutani et al., 1999). The preserved resistance to UVC observed in ADAR1-deficient cells is therefore likely explained by the activity of such factors, which may compensate for subtle post-transcriptional effects on DNA repair–related RNAs. A-to-I RNA editing may broadly influence a wide range of DNA repair pathways, and the combined effects of these alterations are likely reflected in the survival outcomes following exposure to DNA-damaging agents.

In line with our results, previous studies have shown that ADAR1 is crucial for cellular responses to various genotoxic stresses. ADAR1 promotes cell survival following cisplatin treatment through A-to-I editing of the BRCA2 transcript (Liu et al., 2024). Another recent study found that elevated ADAR1 expression confers chemoresistance to 5-fluorouracil and cisplatin by editing the 3′UTR of the stearoyl-CoA desaturase gene transcript (Wong et al., 2023). ADAR1 also exhibits anti-apoptotic effects in response to UV-induced stress (Sakurai et al., 2017). Under CPT treatment, ADAR1 p110 functions non-catalytically by accumulating at R-loops and recruiting DHX9 and DDX21, thereby enhancing ATR activation (Cui H. et al., 2022; Zhang et al., 2023). Our analysis extends this understanding by demonstrating that ADAR1 broadly edits transcripts encoding genome maintenance factors. These observations suggest that the diverse responses to genotoxic agents under ADAR1 deficiency may result from a combination of impaired A-to-I editing across multiple DNA repair-related transcripts and disruption of ADAR1’s non-catalytic roles, such as R-loop resolution. These findings suggest a potential multifaceted role of ADAR1 in genome stability. However, the precise contribution of each isoform still remains unclear. Further investigations, including isoform-specific functional analyses, will be necessary to clarify how ADAR1 regulates genome stability, particularly in relation to transcriptomic remodeling and RNA–protein interactions in DNA repair pathways.

## Data Availability

The datasets generated for this study have been deposited in the DDBJ with accession number PRJDB35672 (https://www.ncbi.nlm.nih.gov/bioproject/PRJDB35672/).

## Abbreviations

m6A, *N* ^6^-methyladenosine; 5-mC, 5-methylcytidine; Ψ, seudouridine; A-to-I, Adenosine-to-Inosine; ADAR, Adenosine Deaminase Acting on RNA; dsRNA, double-stranded RNA; 3′UTRs, 3′ untranslated regions; sgRNA, single-guide RNA; ALT, Alternative lengthening of telomeres; DEG, Differentially expressed genes; DSB, Double-strand break; DDR, DNA damage response; GO, Gene Ontology; IGV, Integrative Genomics Viewer; MEME, Multiple EM for Motif Elicitation; UMI: Unique molecular identifier.
