# Supplementary material for: Epitranscriptome-wide profiling identifies RNA editing events regulated by ADAR1 that are associated with DNA repair mechanisms in human TK6 cells
Source: Front Genet. 2025 Oct 15;16:1663827. doi: 10.3389/fgene.2025.1663827 (PMC12562983; doi:10.3389/fgene.2025.1663827)
Supplement: Supplementary file 3 [file DataSheet1.pdf]

Fig.S1

A

| Sample | Reported A-to-I sites by EpiPlex | Mean A-to-I density in peaks per 100bp | Total theoretical REDIportal sites in EpiPlex peaks | Mean REDIportal site density per 100bp |
|---|---|---|---|---|
| WT rep1 | 71,133 | 7.15 | 163,086 | 17.01 |
| WT rep1 | 66,469 | 7.36 | 150,025 | 17.27 |
| p150KO rep1 | 14,374 | 6.35 | 40,884 | 18.01 |
| p150KO rep2 | 10,159 | 7.84 | 25,191 | 19.56 |
| p150/p110KO rep1 | 4 | 0.6 | 0 | 0 |
| p150/p110KO rep2 | 0 | 0 | 0 | 0 |

B

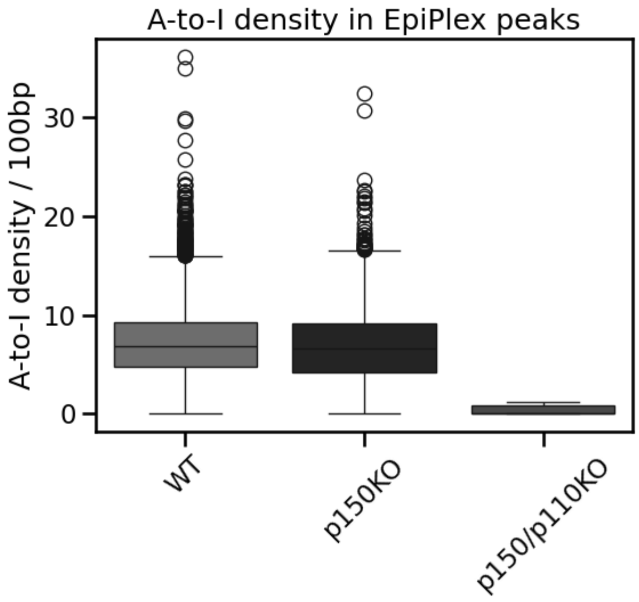


C

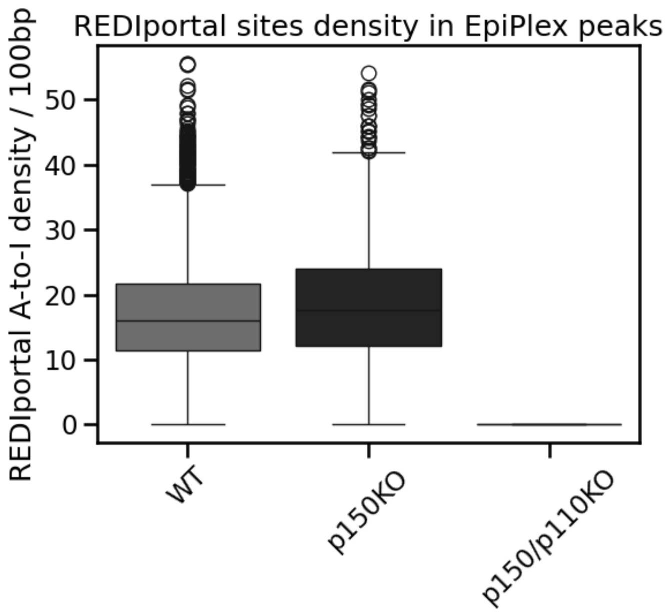


D

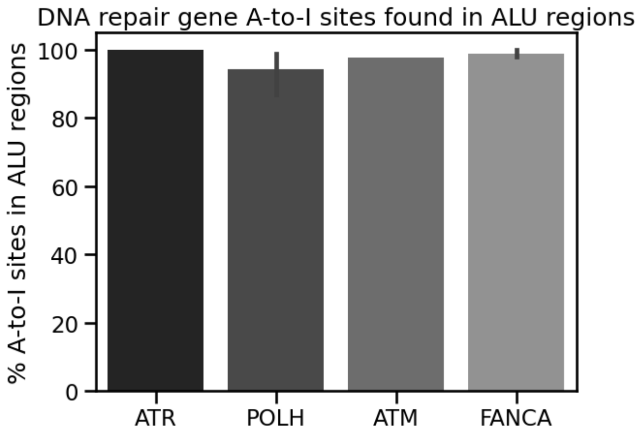


**Figure S1. Summary and distribution of A-to-I RNA editing in TK6 cell phenotypes.**
(A) Summary table of A-to-I counts in REDIportal and EpiPlex samples. Data from two independent clones of each cell type (WT, p150KO, and p150/p110KO cells) are shown. (B) A-to-I densities of TK6 cell phenotypes. Boxplot showing the density of A-to-I editing events per 100 bp within EpiPlex peaks across WT, p150 KO, and p150/p110 KO cells. (C) REDIportal A-to-I site densities of TK6 cell phenotypes. Boxplot showing the density of known A-to-I editing sites (annotated in the REDIportal database) per 100 bp within EpiPlex peaks across WT, p150 KO, and p150/p110 cells. (D) Density of Alu elements in DNA repair genes as annotated by REDIportal A-to-I sites. The only captured ATR inosine peak contains 100% A-to-I sites in Alu elements, while peaks in other DNA repair genes contain >95% Alu elements. Error bars represent the standard error of Alu density in gene's inosine peaks.

Fig.S2

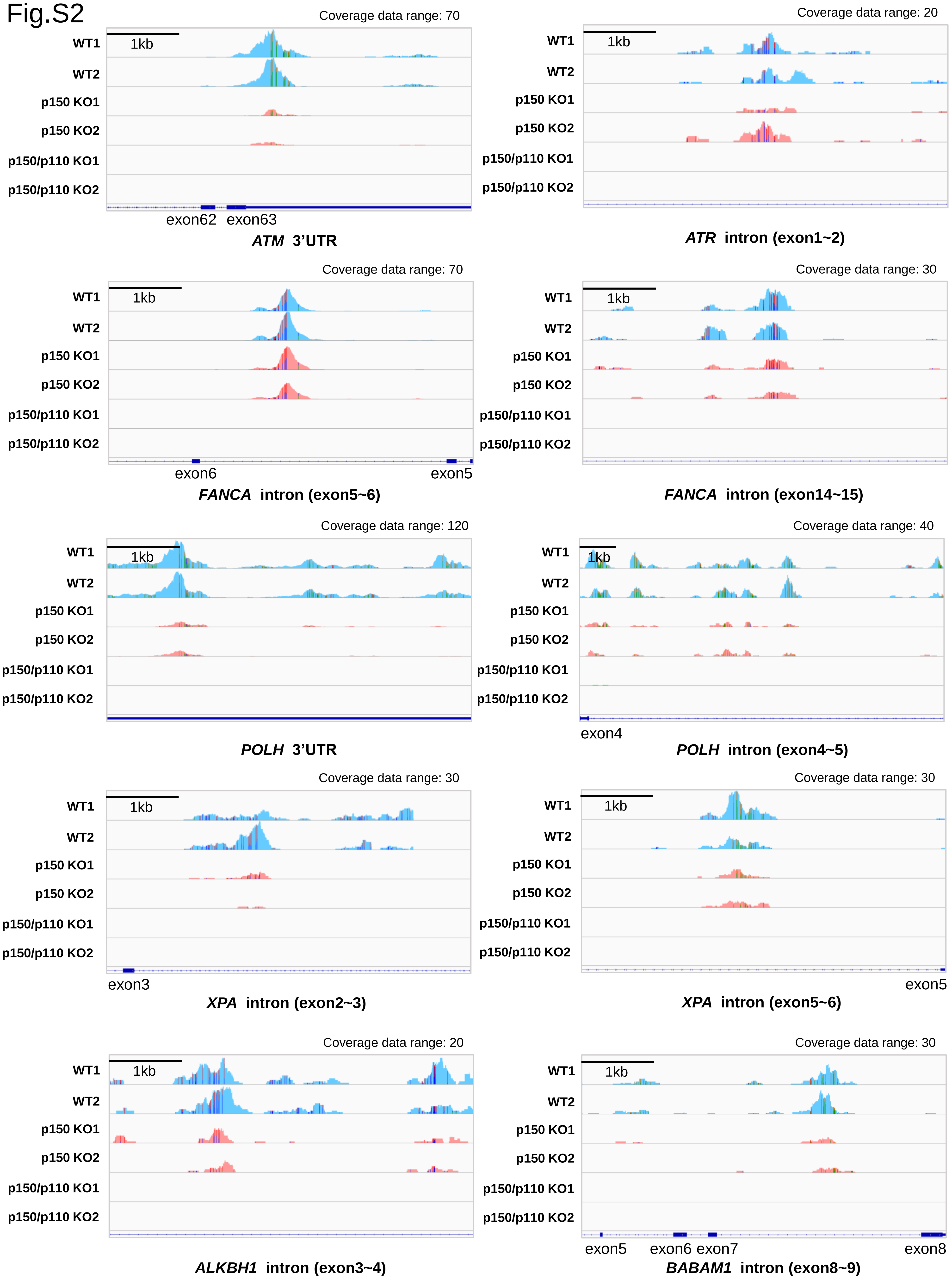

Coverage data range: 70
WT1
WT2
p150 KO1
p150 KO2
p150/p110 KO1
p150/p110 KO2
1kb
exon62 exon63
*ATM* 3'UTR
Coverage data range: 20
WT1
WT2
p150 KO1
p150 KO2
p150/p110 KO1
p150/p110 KO2
1kb
*ATR* intron (exon1~2)
Coverage data range: 70
1kb
exon6
exon5
*FANCA* intron (exon5~6)
Coverage data range: 30
1kb
*FANCA* intron (exon14~15)
Coverage data range: 120
1kb
*POLH* 3'UTR
Coverage data range: 40
1kb
exon4
*POLH* intron (exon4~5)
Coverage data range: 30
1kb
exon3
*XPA* intron (exon2~3)
Coverage data range: 30
1kb
exon5
*XPA* intron (exon5~6)
Coverage data range: 20
1kb
*ALKBH1* intron (exon3~4)
Coverage data range: 30
1kb
exon5 exon6 exon7 exon8
*BABAM1* intron (exon8~9)

Fig.S2 (continued)

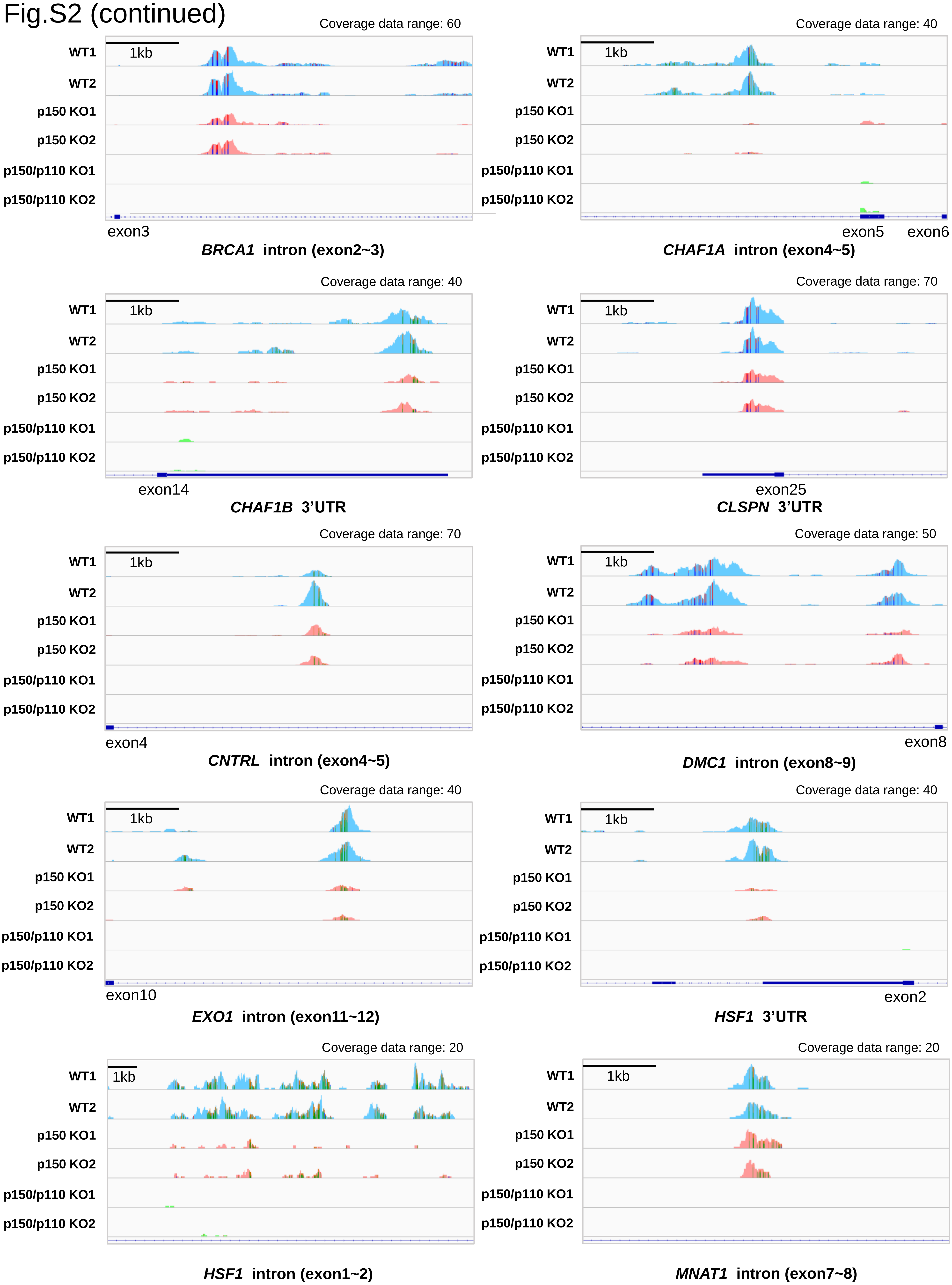

Fig.S2 (continued)

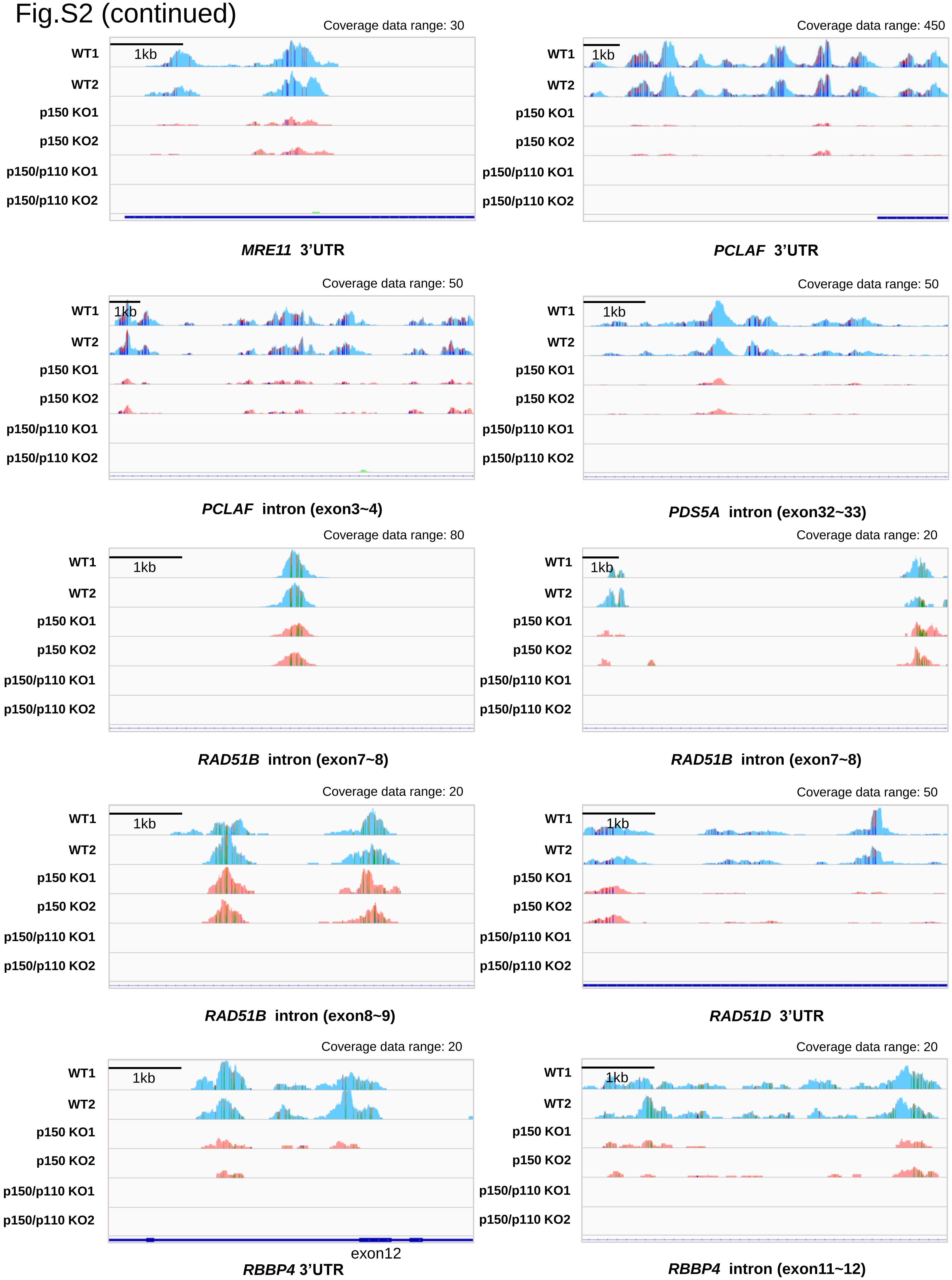

Fig.S2 (continued)

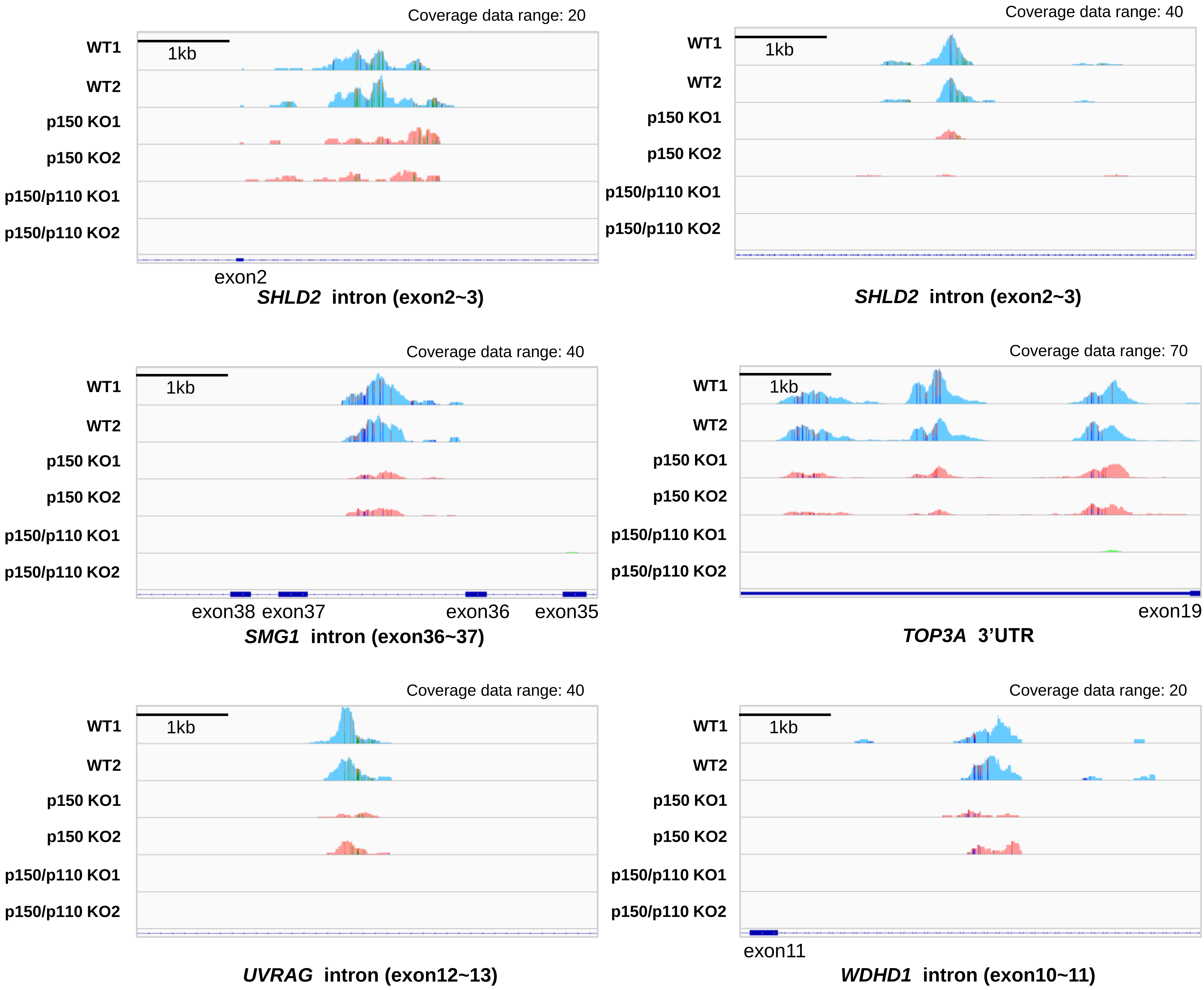


**Figure S2. Visualization of A-to-I RNA editing loci in transcripts of all DNA repair-related genes in wild-type and ADAR1-deficient TK6 cells.** Read coverage profiles are shown for representative DNA repair-associated genes in wild-type (cyan), p150 KO (pink), and p150/p110 KO cells (green). For each cell type, results from two independent clones are presented. The y-axis represents read counts and the x-axis indicates genomic coordinates. The scale bar corresponds to 1 kb. "Coverage data range" in the top-right corner of each panel indicates the maximum coverage value. Colored regions within coverage tracks highlight A-to-I RNA editing sites. Strand-specific colors compositions are shown: green (Adenosine) and orange (Guanosine) for forward reads; red (Thymidine) and blue (Cytidine) for reverse reads, facilitating visual estimation of nucleotide ratios at individual positions.

Fig.S3

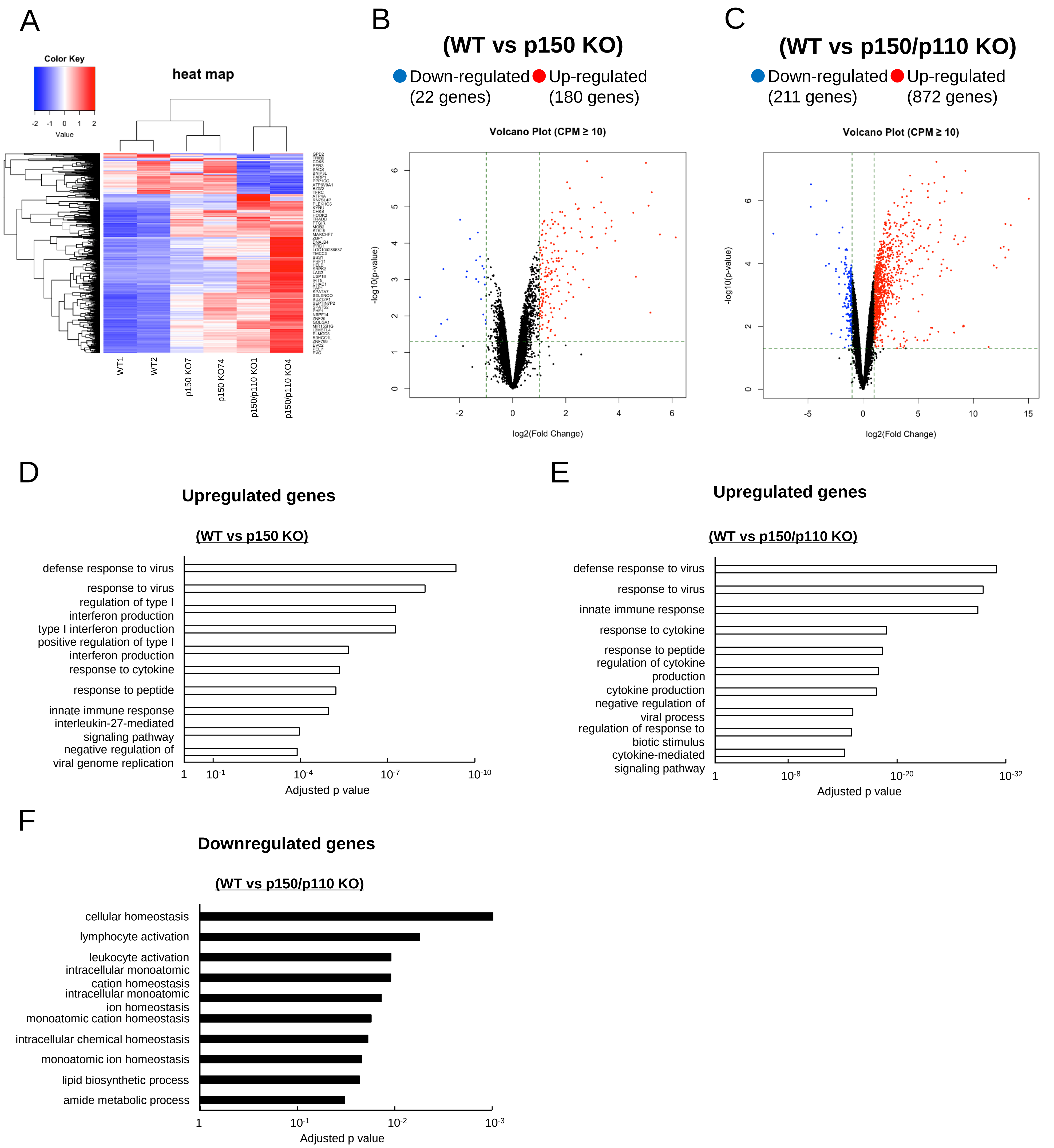


**Figure S3. Differential gene expression between wild-type and ADAR1-deficient TK6 cells.** (A) Heatmap of all differentially expressed genes (DEGs) across WT#1, WT#2, p150 KO#1, p150 KO#2, p150/p110 KO#1, and p150/p110 KO#2 cells. (B) Volcano plot showing DEGs between WT vs p150 KO cells. (C) Volcano plot showing DEGs between WT vs p150/p110 KO cells. (D) GO enrichment analysis of genes upregulated in p150 KO cells compared to WT. (E) GO enrichment analysis of genes upregulated in p150/p110 KO cells compared to WT. (F) GO enrichment analysis of genes downregulated in p150/p110 KO cells compared to WT.
